# pH-dependent genotypic and phenotypic variability in *Oleidesulfovibrio alaskensis* G20

**DOI:** 10.1128/aem.02565-24

**Published:** 2025-03-26

**Authors:** Priya Saxena, Dipayan Samanta, Payal Thakur, Kian Mau Goh, Mahadevan Subramaniam, Brent M. Peyton, Matthew Fields, Rajesh K. Sani

**Affiliations:** 1 Department of Chemical and Biological Engineering, South Dakota School of Mines and Technology 6806 , Rapid City, South Dakota, USA; 2 Data-Driven Material Discovery Center for Bioengineering Innovation, South Dakota School of Mines and Technology 6806 , Rapid City, South Dakota, USA; 3 Faculty of Science, Universiti Teknologi Malaysia, Johor, Malaysia; 4 Computer Science, College of Information Science and Technology, University of Nebraska Omaha 14720 , Omaha, Nebraska, USA; 5 Department of Chemical and Biological Engineering, Montana State University 33052 , Bozeman, Montana, USA; 6 Department of Microbiology and Immunology, Montana State University 123776 , Bozeman, Montana, USA; University of Delaware, Lewes, Delaware, USA

**Keywords:** dissimilatory sulfate reduction, extracellular polymeric substance, energy metabolism, gene regulation, cell morphology, pH homeostasis

## Abstract

Sulfate-reducing bacteria (SRB) exhibit versatile metabolic adaptability with significant flexibility influenced by pH fluctuations, which play a critical role in biogeochemical cycles. In this study, we used a model SRB, *Oleidesulfovibrio alaskensis* G20, to determine the temporal effects of pH variations (pH 6, 7, and 8) on both growth dynamics and metabolic gene expressions. The specific growth rate at pH 6 (0.014 h^−1^) closely matched that at pH 7 (0.016 h^−1^), while pH 8 exhibited a lower growth rate (0.010 h^−1^). Lactate consumption peaked at pH 7 (0.35 mM lactate.h^−1^) and declined at pH 8 (0.09 mM lactate.h^−1^). Significant hydrogen production was evident under both acidic and alkaline conditions. Gene expression studies revealed that ATPases function as proton pumps, while hydrogenases mediate reversible proton-to-hydrogen conversion. Sulfate and energy metabolism act as electron acceptors and donors, while amino acid synthesis regulates basic and acidic amino acids to mitigate pH stress. Downregulation of *FtsZ* at pH 6 suggests impaired division, correlating with slightly longer lengths (~2 µm), while upregulation of divisome proteins at pH 8 suggests efficient division processes, aligning with shorter lengths (~1.8 µm). This study will facilitate the employment of *O. alaskensis* G20 in extreme pH environments, enhancing its effectiveness in optimizing bioremediation and anaerobic digestion processes.

**IMPORTANCE:**

Sulfate-reducing bacteria (SRB) play essential roles in global sulfur and carbon cycling and are critical for bioremediation and anaerobic digestion processes. However, detailed studies on the genotypic and phenotypic responses of SRB under varying pH conditions are limited. This study addresses this gap by examining the pH-dependent genetic and metabolic adaptations of *Oleidesulfovibrio alaskensis* G20, revealing key mechanisms regulating hydrogenase and ATPase activities, cell division, and extracellular polymeric substance formation. These findings provide new insights into how SRB maintains pH homeostasis, showcasing their ability to survive and function in both acidic and alkaline environments. Furthermore, this study reveals critical genetic and phenotypic characteristics that will directly aid to engineer industrial effluent management systems, bioremediation, and dissolved heavy metal recovery. By elucidating the dynamic response of *O. alaskensis* G20 to varied pH environments, the research provides a foundation for enhancing the resilience and performance of SRB-based systems, paving the way for improved environmental and industrial applications.

## INTRODUCTION

Sulfate-reducing bacteria (SRB) are captivating microorganisms that inhabit diverse environments such as marine sediments, wastewater systems, and oil reservoirs, displaying remarkable metabolic versatility and playing crucial roles in global sulfur and carbon cycling (1, 2). The metabolic versatility exhibited by SRB is intricately linked to the diverse environments they inhabit. Therefore, gaining a comprehensive understanding of the governing mechanisms by which pH influences SRB physiology holds particular interest, as it promises to yield valuable insights into their adaptation strategies, metabolic versatility, and intricate ecological interactions. SRB maintains its pH homeostasis by regulating the intracellular pH through various mechanisms, including proton pumps and exchangers, ATPases, and ion channels (3, 4). Alterations in pH homeostasis can exert a substantial influence on the growth, metabolism, biochemical processes, and physiological functions of SRB (5).

In the realm of microbiology, pH exerts multifaceted effects on bacterial metabolism, influencing cellular growth patterns, activation of stress metabolism, and alteration in extracellular enzyme activities (6, 7). Additionally, pH plays a crucial role in determining the concentration of nutrients available to bacteria by regulating processes such as nutrient dissolution, precipitation, and geochemical interactions, which collectively affect nutrient solubility, bioavailability, and distribution in the surrounding environment (4). Researchers have noted that extracellular enzymes involved in the degradation of organic substrates can be particularly susceptible to pH changes, with increasing pH levels resulting in reduced enzymatic capacity (8).

SRB is equipped with metabolic pathways that can help to regulate the elevated pH conditions by controlling the production of protons and hydroxide ions through their metabolic processes, including lactate/acetate oxidation and sulfate reduction (4, 9, 10). Nevertheless, it has been observed that SRB possesses the capability to produce hydrogen gas through their high hydrogenase activity while using lactate, ethanol, formate, and butyrate as electron donors, particularly in sulfate-limited environments (11). Simultaneously, they also have the unique ability to use hydrogen gas (H_2_) as an electron donor to reduce sulfate (SO_4_^2−^) to sulfide (S^2−^) through the activity of the enzyme hydrogenase (12). Heidelberg et al. (13) performed whole-genome sequencing and revealed novel hydrogenase isozymes, such as cytoplasmic isozymes in *Desulfovibrio vulgaris*, providing convincing evidence for the essential role of these isozymes in energy conservation via the “hydrogen-cycling” model (13). The identification of a considerable number of formate dehydrogenases (FDHs) also indicates a secondary system of chemiosmotic energy conservation through the diffusion of formic acid from the cytosol and its subsequent oxidation in the periplasm (13). This ability to modulate pH levels enables SRBs to maintain optimal conditions for their growth and survival in various environments, ranging from marine sediments to anaerobic wastewater treatment systems (14). Contrarily, Kikot et al. (15) demonstrated that modifications in pH levels had a substantial impact on sulfate reduction within the SRB community. In contrast, the existence of heavy metals did not cause any significant effect on this process (15). Gutierrez et al. found that alkaline pH levels (8.6 and 9.0) significantly reduced the activity of SRB, leading to a notable decrease in sulfide production in anaerobic sewer biofilms (16). Thus, it is important to consider the effects of environmental pH when studying the activity and ecology of SRB in natural and engineered systems.

Until now, the temporal pH adjustment mechanisms in *Oleidesulfovibrio alaskensis* (OA G20) have not been explored, leaving a critical knowledge gap regarding the key genes and associated pathways responsible for this adaptation. In this study, we used *O. alaskensis* G20 as a model neutrophilic SRB to investigate the mechanisms of pH homeostasis in both acidic and alkaline environments. Our study explores the genetic foundations governing metabolic pathways within *O. alaskensis* G20, identifying crucial genes and their expression in response to pH regulation. Additionally, our study examines the formation of extracellular polymeric substances (EPS), along with associated phenotypic changes/cell morphology (size), at varied pH conditions. This study will aid several strain engineering efforts aimed at enhancing resilience to pH stress, ensuring robust performance in industrial effluent management systems. For instance, *O. alaskensis* G20’s ability to maintain activity under varied pH conditions can be leveraged for enhanced heavy metal precipitation in wastewater treatment, where fluctuating pH is a challenge. Similarly, the metabolic insights gained could optimize bioreactors for dissolved metal recovery by fine-tuning operational pH to maximize SRB-mediated sulfide production and metal precipitation.

## MATERIALS AND METHODS

### Bacterial strain and growth conditions

The *O. alaskensis* G20 strain used in this study was obtained from our laboratory collection (South Dakota Mines), originally sourced from previous work conducted by Sani et al. (17). Anaerobic batch cultivation of *O. alaskensis* G20 was conducted in 125 mL serum bottles sealed with rubber septa and aluminum caps. Lactate C medium was used as the growth medium, containing 60 mM sodium lactate as the electron donor and 50 mM sodium sulfate as the electron acceptor (18). The pH range for the experiment was adjusted from 4 to 8 by the addition of 5N hydrochloric acid for pH reduction and 5 M sodium hydroxide for pH elevation. Serum bottles containing 100 mL media were autoclaved at 121°C for 15 minutes. Following autoclaving, the serum bottles were deoxygenated by purging with filter-sterilized ultrapure nitrogen for 20 minutes. A 5% (vol/vol) inoculum of *O. alaskensis* G20 seed culture (OD_600_ = 0.178), prepared using the same lactate C media composition and procedure mentioned above, was added to the serum bottles using a sterilized syringe (10 mL, 21-gauge needle). The seed culture was sourced from a 40% glycerol stock suspension stored at −80°C, and prior to inoculation, the seed culture was purged with filter-sterilized ultrapure nitrogen for 1 h to remove any hydrogen sulfide (H_2_S) present. Throughout the experimental period, the culture-containing serum bottles were incubated at 30°C and subjected to continuous shaking at 125 rpm. The experiment was performed in triplicate and repeated to ensure the reliability of the results.

### Measurement of growth statistics

The growth of *O. alaskensis* G20 cells under each pH condition was assessed using an absorbance spectrophotometer (BioTek Instruments, Winooski, VT, USA) at 600 nm. Measurements were recorded every 24 h over a span of 6 days. The growth statistics for all samples were analyzed using an exponential fitting curve (fitting in Polymath software), achieving a goodness of fit (*R*^2^) value of 0.90% ± 3%. The maximum specific growth rates (*µ*_max_) were calculated using the *N*_*t*_ = *N*_0_*exp(*µ*t), where *N*_*t*_ and *N*_0_ represent the initial and final cell numbers, assuming the increase in OD values is directly proportional to the increase in cell number in a closed system, and *µ* denotes the specific growth rate (19).

### Measurement of lactate consumption using ion-exchange chromatography

The lactate consumption was measured using ion-exchange chromatography (Dionex Aquion, Thermo Fisher Scientific, Waltham, MA, USA), equipped with a conductivity detector and Dionex IonPac AS22 column. Bacterial culture (1.5 mL) was transferred by syringe to 2 mL Eppendorf tubes from each of the serum bottles. Then, centrifugation was performed at 6,500 × *g* for 10 minutes to remove any cells, and the supernatant was further filtered using 0.22 µm filters to ensure cell-free supernatant. The lactate standards were prepared using the 1 M stock solution of sodium lactate. To prepare the stocks and standards, high performance liquid chromatography (HPLC) grade water was used. The known concentrations were adjusted as 0, 5, 10, 25, 50, and 75 mM sodium lactate.

### Total RNA extraction

Total RNA was isolated from the control and test samples under variable pH (6, 7, and 8) by collecting 10 mL of liquid culture from the serum bottles during the growing and stationary phases. The OD_600_ of *O. alaskensis* G20 cultures on days 2 and 4 corresponds to the exponential and stationarity phase, respectively. Each 10 mL culture was centrifuged at 6,500 × *g* for 10 minutes at 4°C, and the cell pellets were collected. The pellets were washed three times with 1 mL of anaerobic phosphate-buffered saline (PBS; composition: 1.76 mM KH_2_PO_4_, 10.1 mM Na_2_HPO_4_, 2.60 mM KCl, 1.37 mM NaCl, and pH 7.4) and subsequently transferred to sterile 2 mL microcentrifuge tubes for further extraction. All steps were conducted within an anaerobic chamber (COY Lab Products, Grass Lake, MI, USA) to maintain strict anoxic conditions throughout the experimental procedure. Total RNA was extracted using a complete DNA and RNA Purification Kit following the manufacturer’s instructions (Lucigen, Middleton, WI, USA) and eluted in 20 µL Tris-EDTA buffer. RNA purity was accessed using Nanodrop UV-Vis spectrophotometer (Thermo Fisher Scientific, Waltham, MA, USA). The RNA concentrations were further confirmed using the Qubit RNA assay kit and Qubit 3.0 fluorometer (Thermo Fisher Scientific, Waltham, MA, USA). Finally, RNA integrity was analyzed by electrophoresis using the Bioanalyzer 2100 system (Agilent Technologies, Santa Clara, CA, USA).

### cDNA synthesis and quantitative real-time PCR

To unravel the genetic variations linked to cell survival under acidic and alkaline conditions, key genes involved in pH homeostasis were specifically targeted for analysis using RT-PCR. Initially, the cDNA of each sample was synthesized from 1.5 µL of the extracted total RNA using the QuantiTect Reverse Transcription Kit (Qiagen, Germantown, MD, USA). RT-qPCR was then performed in a QuantStudio 3 Real-Time PCR system (Model #A28132, Thermo Fisher Scientific, Waltham, MA, USA) using the Maxima SYBR Green/ROX qPCR Master Mix (Thermo Fisher Scientific, Waltham, MA, USA) in 0.2 mL PCR tube strips (Thermo Fisher Scientific, Waltham, MA, USA). The gene-specific primer sequences and NCBI accession IDs are listed in Table S1 (GenBank genome accession no. GCA_000012665.1). The 16S rRNA gene was used as an internal control for PCR amplification and data normalization. Relative expression levels between control (pH 7) and test samples (pH 6 and 8) were determined using the 2−ΔΔCT method. Gene expression data are reported as base-2 logarithms of the 2−ΔΔCT values, expressed as log2FC (20). All experiments were performed in triplicate with independent samples, and mean values along with SEs were calculated.

### Measurement of hydrogen

Hydrogen generation and consumption in the headspace of each batch culture were evaluated using a gas chromatograph (Model #8610C, SRI Instruments, Torrance, CA, USA) equipped with a thermal conductivity detector and 3' × 1/8" molecular sieve 5A packed column (Model# 8600-PK2A, SRI Instruments, Torrance, CA, USA) (21). Given the substantial difference in thermal conductivity between argon (0.018 W/mK) and the gases measured (hydrogen, 0.0343 W/mK) at 300 K and 1 atm, argon was employed as the carrier gas (supplied at 40 psi), and the chromatograph was maintained at a constant pressure of 14 psi. The oven temperature was incrementally adjusted from 150°C to 250°C in 10°C steps according to Samanta et al. (22). The hydrogen peak was detected at a retention time of 0.72 minutes, and the corresponding peak areas were recorded. The peak areas were calculated post-run using the “immediate integrate” option in the event window of the PeakSimple489 software, compatible with the 8610C model. These peak areas were compared to a calibration curve of known concentrations to determine the hydrogen concentration. Hydrogen concentration measurements were taken every 24 h over a period of 6 days.

### Cell morphology visualization using scanning electron microscope

The cell morphology of planktonic cells at pH 6, 7, and 8 was examined using a field-emission scanning electron microscope (FESEM, Zeiss Supra40 variable pressure). Planktonic culture samples (1 mL) were withdrawn from respective serum bottles on experimental days 2 and 4, followed by centrifugation at 3,250 × *g* for 5 minutes. The resulting cell pellet was then washed with PBS at pH 7.2. After washing, the cell pellet was fixed by immersion in a solution containing 0.1 M sodium cacodylate buffer and 2% glutaraldehyde overnight at 4°C, followed by an additional hour at room temperature. Subsequently, the cells underwent sequential dehydration using ethanol (50%, 75%, 95%, and 100%) for 30 minutes each and dried overnight in a desiccator. The SEM images were obtained using secondary electron imaging operated at an accelerated voltage of 3 kV. The images were processed with magnifications of 8,000× and 12,000× (scaled at 4 and 2 µm, respectively). The cell lengths (cell count, 3 ≤ *n* ≤ 7) were measured along a one-dimensional linear axis.

### Extraction and quantification of crude exopolysaccharides

On days 2 and 4 of the experimental period, 10 mL of the culture broth from each serum bottle was aseptically transferred to 50 mL falcon tubes and subsequently centrifuged at 6,500 × *g* for 10 minutes to separate the cells from the broth. To ensure the complete removal of cells, the resulting supernatant was additionally filtered using 0.22 µm syringe filters. The cell-free supernatant was then combined with an equal volume of chilled absolute ethanol (−20°C), which was added dropwise while continuously stirring in an ice bath. Following this, the alcoholic solution was placed at −20°C overnight and subsequently centrifuged at 10,000 rpm for 40 minutes. The resulting pellet was dissolved in 1 mL of deionized water (23).

Total carbohydrate content was assessed using the phenol-sulfuric acid method, where glucose was used as the standard (24). Protein content was estimated using the Qubit Protein Broad Range (BR) assay kit and a Qubit 3.0 fluorometer (Thermo Fisher Scientific, Waltham, MA, USA). Nucleic acid content was measured using a Nanodrop UV-Vis spectrophotometer (Thermo Fisher Scientific, Waltham, MA, USA) at a wavelength of 260 nm. The same procedure was performed on the uninoculated medium as a control. The carbohydrate, protein, and nucleic acid contents of the EPS were calculated by subtracting the corresponding values obtained from the blank medium.

## RESULTS AND DISCUSSION

### Effect of pH on the growth of *O. alaskensis* G20 cells and lactate consumption

**Fig 1:**
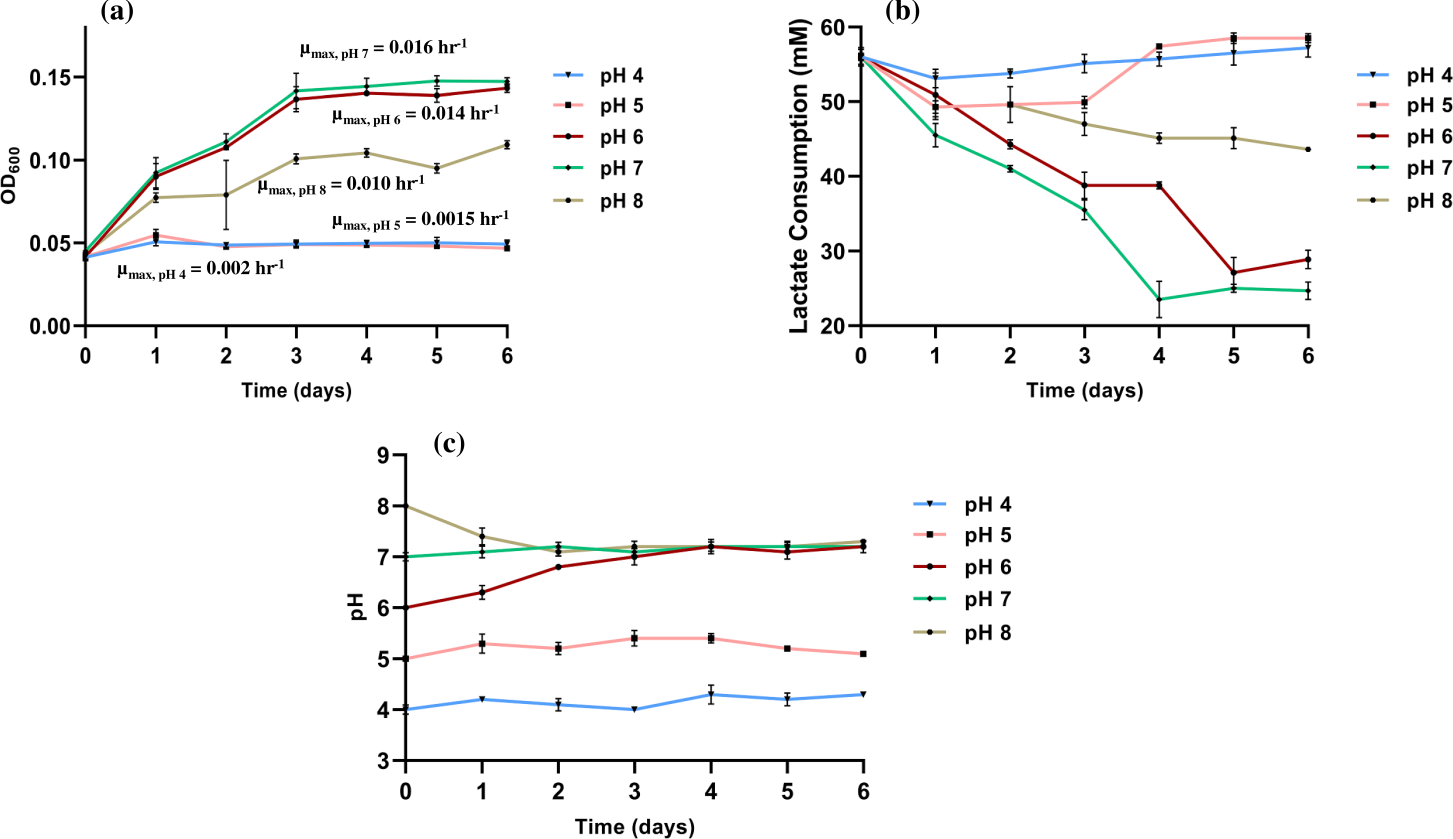
Effect of pH on the (a) *O. alaskensis* G20 growth, (b) lactate consumption, and (c) pH variation.

The growth of *O. alaskensis* G20 cells exhibited significant variations in response to changes in pH levels (Fig. 1a). Among the pH levels tested, pH 7 and 6 demonstrated the maximum growth, exhibiting a *µ*_max_ of 0.016 h^−1^ and 0.014 h^−1^, respectively. In contrast, the *µ*_max_ at pH 8 was lower, measuring 0.010 h^−1^. No significant growth was observed at pH 4 (*µ*_max_ = 0.002 h^−1^). The variations in *µ*_max_ between pH 4 and 5 (*µ*_max_ = 0.0015 h^−1^) were minimal, suggesting a similar inhibitory effect on cell growth. These results are in agreement with previous research by Kushkevych et al., who observed inhibition of *Desulfovibrio piger* Vib-7 growth at pH values less than 5 (25). It has been reported that *D. vulgaris* can survive in acidic conditions with a pH of 4, which can lead to increased rates of corrosion when grown in the presence of a metal surface (26). We compared the *µ*_max_ values with the reported literature under other stress-related environments. A study by Thakur et al. reported the *µ*_max_ of *O. alaskensis* G20 under varying copper stress conditions to be between 0.010–0.012 h^−^¹, compared to 0.015 h^−^¹ in the control (pH 7.2, lactate-C media) (27). Similarly, Williamson et al. observed *µ*_max_ values of 0.06 h^−^¹ at 0.1 MPa and 0.04 h^−^¹ at 14 MPa under pressure stress conditions in pH 7.4 lactate/sulfate media (28). This suggests that the cellular growth of *O. alaskensis* G20 is highly sensitive to environmental factors, as evidenced by previous studies on copper and pressure stress, and further highlighted in this study focusing on pH.

The observed lactate concentration (Fig. 1b) exhibited a direct association with the growth of *O. alaskensis* G20 cells, in line with the widely recognized principle that higher cell numbers correspond to increased substrate consumption. During anaerobic lactate oxidation, a stoichiometric ratio of two moles of lactate is required to react with one mole of sulfate, resulting in the production of one mole of H_2_S, two moles of an organic compound (typically acetate), two moles of carbon dioxide, and two molecules of water (29). The primary metabolic reaction can be expressed as:


$$2\;CH_{3}CHOHCOOH+H_{2}SO_{4}⟶2\;CH_{3}COOH+2\;CO_{2}+H_{2}S+2\;H_{2}O.$$

In this study, the highest lactate consumption rate was observed at pH 7, with a rate of 0.35 mM lactate.h^−1^. This was followed by pH 6, where lactate was consumed at a rate of 0.17 mM lactate.h^−1^. In contrast, at pH 8, the lactate consumption rate decreased to 0.09 mM lactate.h^−1^. Notably, pH 4 and pH 5 showed a negligible reduction in lactate concentration, reflecting the lack of measurable cell proliferation at these pH levels.

The temporal change in pH over the 6-day experimental period is shown in Fig. 1c. It is noteworthy that pH 8 consistently approached near pH 7 by day 2 of the experiment and maintained a pH of approximately 7 throughout the remaining 4-day period. Similarly, pH 6 reached pH 7 on day 3 and continued to exhibit growth at this pH. These findings indicate that *O. alaskensis* G20 cells display a preference for pH 7 and adapt to this condition for optimal growth. The distinct lack of growth at pH 4 suggests that extreme acidic conditions are highly unfavorable for the growth of *O. alaskensis* G20 cells. A similar pH shift was observed in the case of *D. vulgaris* under alkaline stress (30). However, the question arises: why do *O. alaskensis* G20 cells struggle to thrive at pH 8 compared to pH 6, especially considering they reach approximately pH 7 more rapidly than pH 6? This warrants a detailed exploration of the pH-dependent genotypic behavior within *O. alaskensis* G20 cells, including the underlying mechanisms governing pH sensitivity and homeostasis maintenance.

Both H_2_CO_3_ and H_2_S act as weak acids, with dissociation equilibria that significantly influence environmental pH (31–33). The dissociation of H_2_CO_3_ into bicarbonate (HCO_3_^−^) and protons (H^+^) and of H₂S into bisulfide (HS^−^) directly affects proton availability in the environment (Table 1), which in turn impacts pH stabilization. Under alkaline conditions (e.g., pH 8), the dissociation of both H_2_CO_3_ and H_2_S is enhanced, resulting in increased concentrations of HCO_3_^−^ and HS^−^ (34). This dissociation releases protons, effectively lowering the pH and driving the environment toward neutrality. Conversely, under acidic conditions (e.g., pH 6), the equilibrium shifts toward the protonated forms, H_2_CO_3_ and H_2_S, which consume protons during their formation (34). This consumption raises the pH, again pushing the environment toward a neutral range. These inherent buffering effects of weak acids ensure that SRB can maintain a stable pH around neutral values without requiring extensive cellular energy expenditure (35, 36). At pH 7, the pKa values of H_2_CO_3_ (6.4) and H_2_S (7.0) result in approximately equal proportions of their protonated (H_2_CO_3_ and H_2_S) and deprotonated (HCO_3_^−^, HS^−^) forms (37, 38). There is no net proton consumption or production because the medium’s buffering capacity ensures equilibrium between the protonated and deprotonated forms of these acids. The balance of reactants and products maintains the pH near neutrality, consistent with the observed pH stabilization at ~7.3 in the experimental data.

**TABLE 1:** Stoichiometric balance of reactants and products at different pH levels

pH level	Reactants	Products	Proton effect
pH 6	2 CH_3_CHOHCOO^−^ + SO_4_^2−^ + 2 H^+^	2 CH_3_COO^−^ + 2 H_2_CO_3_ + H_2_S	Net proton consumption
pH 7	2 CH_3_CHOHCOO^−^ + SO_4_^2−^	2 CH_3_COO^−^ + HCO_3_^−^ + H_2_CO_3_ + HS^−^ + H_2_S	No net proton flow
pH 8	2 CH_3_CHOHCOO^−^ + SO_4_^2−^	2 CH_3_COO^−^ + 2 HCO_3_^−^ + HS^−^ + H^+^	Net proton production

Beyond the intrinsic role of buffers in pH homeostasis, changes in the expression of genes associated with metabolic pathways have also been reported in earlier studies (30, 39–41). This study further explores pH-associated gene expression in *O. alaskensis* G20, as discussed below.

### Variation in gene expression due to acidic and alkaline pH exposure

Neutrophilic bacteria rely on maintaining a compatible cytoplasmic pH between 7.4 and 7.8 to thrive under both acidic and alkaline conditions (42). Consistently, as described above, it was observed that *O. alaskensis* G20 cells managed to maintain a pH of approximately 7.3 throughout the experimental period after adapting from pH 6 and pH 8. Hence, the findings suggest that *O. alaskensis* G20 cells possess robust pH homeostasis mechanisms that are crucial in maintaining the optimal function and structural integrity of cytoplasmic proteins, thereby facilitating the growth of bacteria. Elevated pH levels pose significant stress to the cell, resulting in cytoplasmic alkalinization, reduction of membrane potential, and potential damage to proteins (43). To overcome these challenges, the cell implements various strategies to mitigate the detrimental effects of elevated pH. These include the active pumping of protons into the cytoplasm, the importation or synthesis of compounds that acidify the cytoplasm, and the activation of systems involved in protein repair or degradation (42). Similarly, cells employ reverse mechanisms to mitigate cell acidification during lowered pH conditions. These mechanisms include the efflux of protons from the cytoplasm and the induction of cytoplasmic alkaline metabolic synthesis (41). Interestingly, Yu et al. found that 57 genes, mainly related to energy metabolism, cell membrane, and protein translation, were responsible for cell survivability in *D. vulgaris* under acidic pH conditions (39). In a related study, researchers investigated the impact of alkaline stress on *D. vulgaris*, in which the mutation studies showed that the maintenance of alkaline pH homeostasis involved specific components, such as the sodium/proton antiporter NhaC-2, tryptophanase A, and two putative regulators/histidine kinases (HKs; DVU0331 and DVU2580) (30).

**Fig 2:**
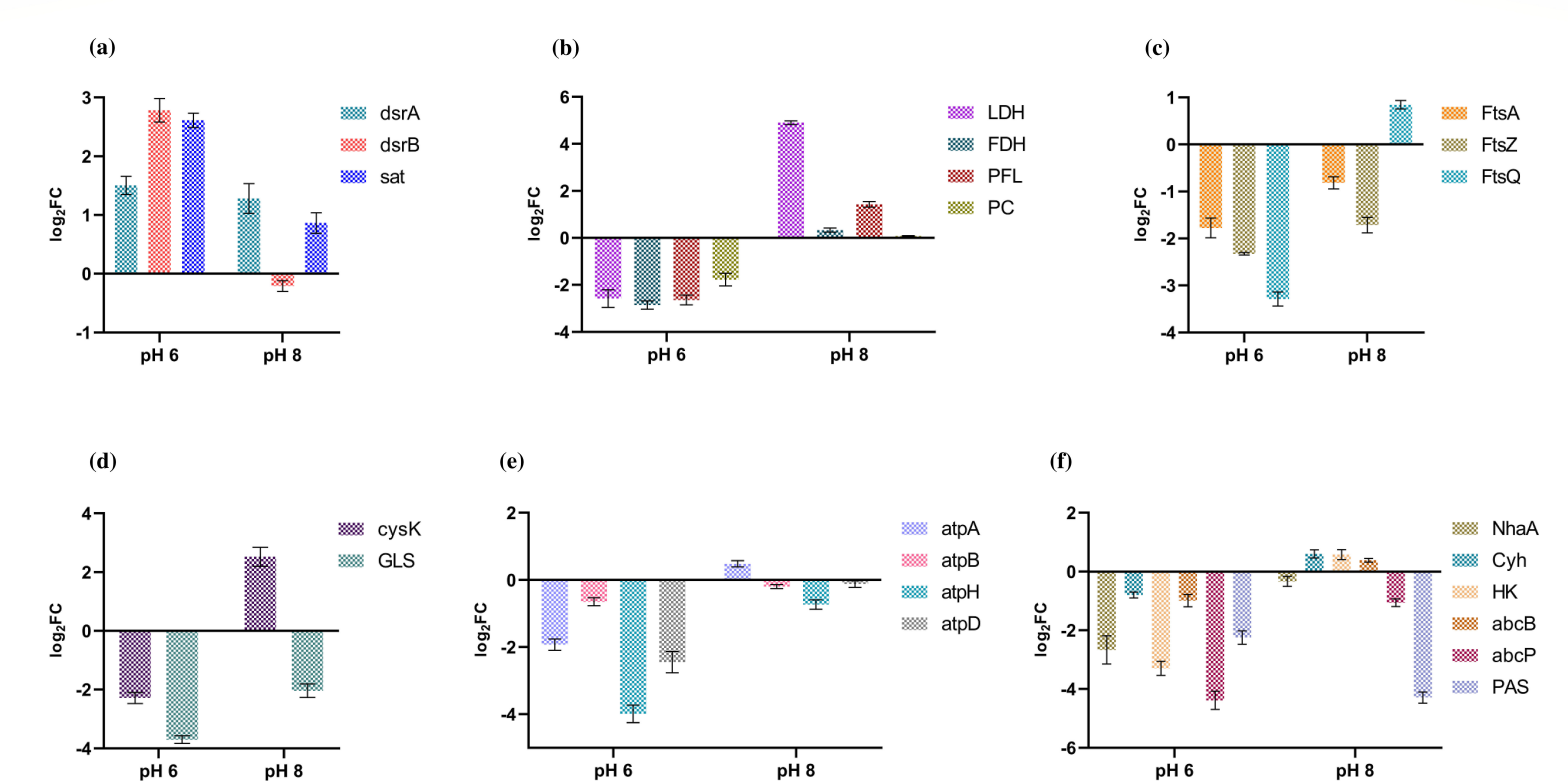
Gene expression profiles for pH 6 and pH 8 at the growing phase with respect to control (pH 7). (a) DSR, (b) central carbon metabolism, (c) cell division coordination, (d) amino acid synthesis, (e) *F*_0_*–F*_1_ ATPases, and (f) transporters.

**Fig 3:**
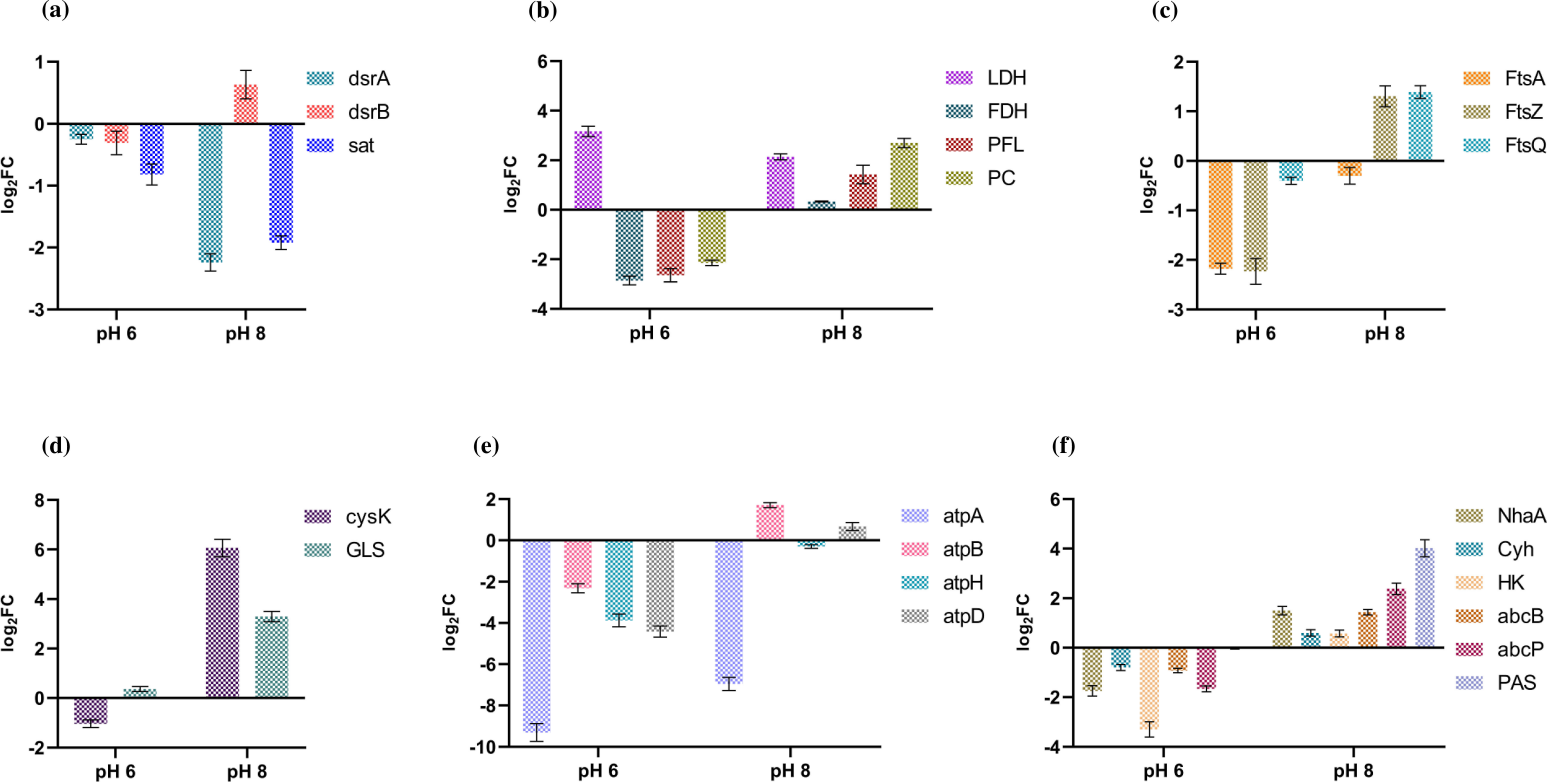
Gene expression profiles for pH 6 and pH 8 at the stationary phase with respect to control (pH 7). (a) DSR, (b) central carbon metabolism, (c) cell division coordination, (d) amino acid synthesis, (e) *F*_0_*–F*_1_ ATPases, and (f) transporters.

Gene expression profiles related to key metabolic processes, including (i) dissimilatory sulfate reduction (DSR), (ii) hydrogenases, (iii) central carbon metabolism, (iv) cell division coordination, (v) amino acid synthesis, (vi) *F*_0_*–F*_1_ ATPases, (vii) transporters, and (viii) polysaccharide synthesis (PS), are depicted for the growing phase in (Fig. 2) and the stationary phase in Fig. 3. The selection of these genes was based on their essential roles in metabolism and relevance to pH-related studies reported in the literature. The study examined both growing and stationary phases to investigate genetic regulation during growth adaptation and pH shift. The gene expression data related to hydrogenases and polysaccharide synthesis are shown in later sections (see “Hydrogenases” and “Quantification of extracellular polymeric substances under variable pH,” respectively).

#### Dissimilatory sulfate reduction

The DSR metabolic pathway involves a series of enzymatic steps to facilitate the process of sulfate reduction. The pathway begins with the activation of free sulfate molecules near bacterial cells, a process facilitated by the enzyme sulfate adenylyltransferase (ATP-sulfurylase). This activation leads to the production of adenosine-5-phosphosulfate (APS) and pyrophosphate (44). Since the reduction potential of sulfate/sulfite is highly negative, it cannot be directly reduced by primary intracellular electron mediators like NADH or ferredoxin (45). In the next step, the enzyme APS reductase reduces cytoplasmic APS, converting it to sulfite/bisulfite and AMP (46). Subsequently, the dissimilatory sulfite reductase (DsrAB) enzyme catalyzes the reduction of sulfite to form a trisulfide intermediate known as DsrC. Finally, the trisulfide intermediate is further reduced to sulfide, along with the production of reduced DsrCr, by the membrane-bound DsrMKJOP complex (47). In DSR, the electron flow encompasses a cascade of redox reactions, whereby electrons are transferred from an electron donor to sulfate, resulting in the production of H_2_S (48). The dissociation of H_2_S in the aqueous phase is influenced by pH levels. At different pH levels, H_2_S can exist in different forms due to the ionization of the compound. The dissociation of H_2_S can be described by the following equilibrium reaction (49):

$H_{2}S\overset{}{⇌}H^{+}+HS^{-}\overset{}{⇌}2H^{+}+S2^{-}$ (pKa = 7 [50]).

At pH 6, high H^+^ levels favor undissociated H_2_S (25, 51). At pH 8, high OH^−^ promotes H₂S dissociation, reducing its toxicity (52).

Our analysis of gene expression data (log2FC), specifically focusing on the DSR genes (*sat*, *dsrA*, and *dsrB*), revealed significant variations in response to acidic (pH 6) and alkaline (pH 8) conditions compared to neutral conditions (pH 7). During the growing stage, we observed an upregulation of *sat* (log2FC = 2.8) and *dsrAB* (log2FC, dsrA = 1.6; dsrB = 2.9) genes in pH 6, and a similar upregulation of *dsrA* (1.2) was observed in pH 8. The upregulation of *dsrB* (0.3) in pH 8 was insignificant. These gene expression patterns align with the lactate consumption data, which indicated higher lactate consumption at pH 6 compared to pH 8. Consequently, based on the stoichiometry of the reaction, it can be inferred that more H_2_S would be formed at pH 6, where lactate consumption is higher. Importantly, at pH 6, H_2_S would be predominantly present in its undissociated form. Conversely, at pH 8, lower lactate consumption results in reduced H_2_S production, and thus, H_2_S would be available in a more dissociated form, where the presence of H^+^ ions helps to balance out environmental OH^−^ ions, effectively neutralizing the pH to approximately 7. During the stationary phase, the cell cultures at pH 6 and pH 8 transitioned toward a neutral pH state. In this stage, the downregulation of *dsrAB* gene expression was observed. Interestingly, there was no significant decline in lactate consumption observed after day 4. As a result, there was limited electron transfer involving lactate as a donor. Consequently, sulfate reduction was not significant under these conditions. The observed downregulation of *dsrAB*, coupled with the diminished lactate consumption, suggests a decrease in the activity of dissimilatory sulfate reduction during the stationary phase at pH 6 and pH 8.

#### Hydrogenases

Hydrogenases are pivotal enzymes involved in hydrogen metabolism, facilitating the reversible conversion between molecular hydrogen (H_2_) and protons (H^+^) through catalytic reactions. The catalytic mechanism of hydrogenases entails the binding and activation of H_2_, followed by its subsequent splitting into protons and electrons (53). The protons can be used in diverse metabolic pathways, while the electrons can be transferred to electron carriers like NAD^+^/NADH or ferredoxin, participating in various redox reactions and energy production processes (54). Notably, the reaction catalyzed by hydrogenases can be reversible, enabling the production of hydrogen when appropriate conditions are met (55). Therefore, expression studies on hydrogenases are crucial to understand the modulation of the proton pump under varying pH conditions. In this study, we focused on NiFeSe and *hyd* hydrogenases to investigate their expression profile in response to pH 6 and pH 8 with respect to pH 7. Caffery et al. conducted a study focusing on *D. vulgaris*, revealing a significant correlation between hydrogenases expression and the concentration of H_2_ in its environment. Their findings suggest that as the H_2_ concentration decreases, the expression of NiFeSe increases, with a notable shift observed when comparing lower (5%) to higher (50%) H_2_ concentrations. Conversely, the expression pattern of *hyd* operates in the opposite direction, demonstrating a reverse relationship with H_2_ concentration (56).

**Fig 4:**
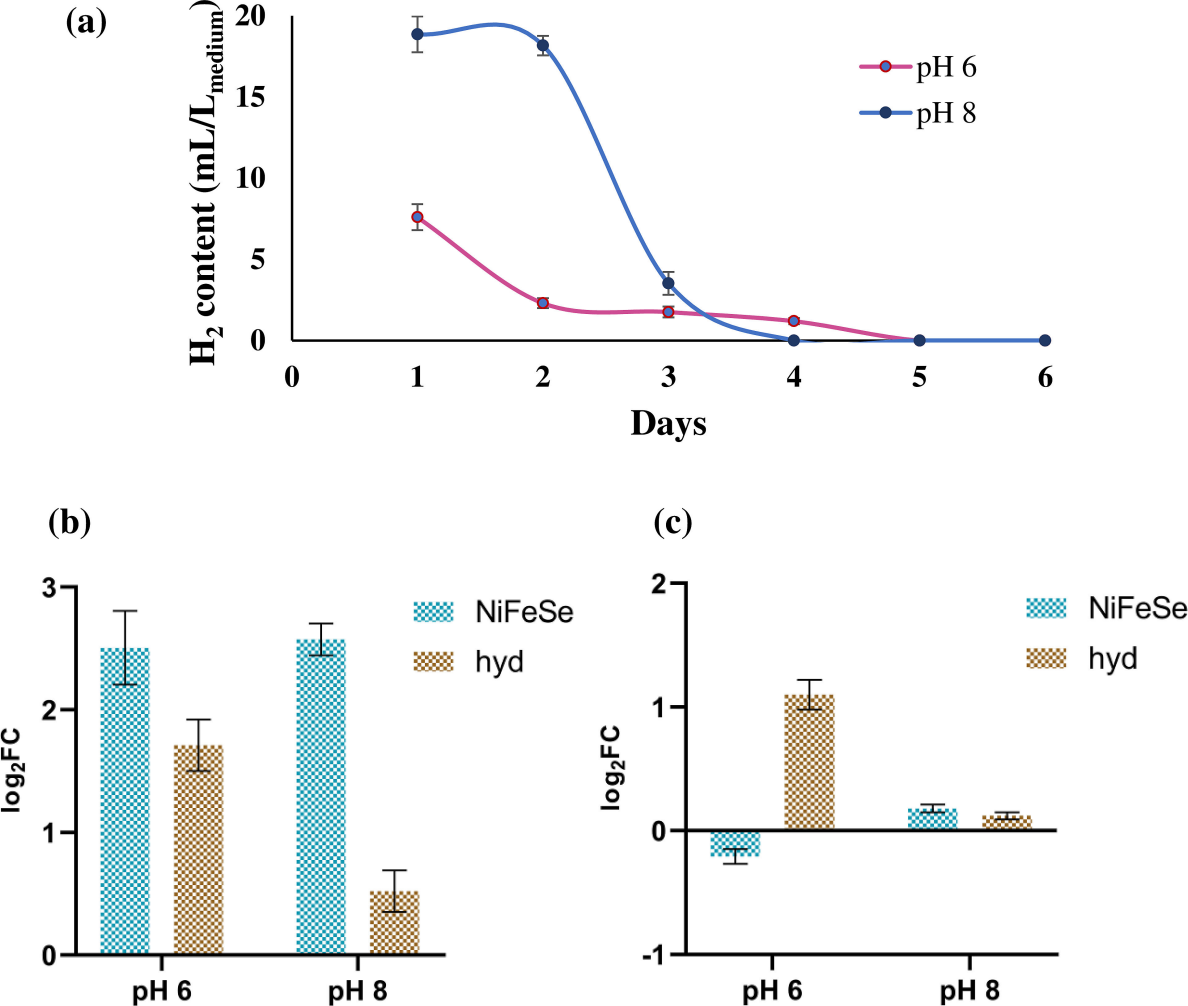
(a) Temporal profile of headspace hydrogen content at pH 6 and pH 8, and gene expression profiles of hydrogenases during (b) growing phase and (c) stationary phase of *O. alaskensis* G20 with respect to pH 7.

As demonstrated in Fig. 4, we observed distinct patterns of H_2_ production by *O. alaskensis* G20 cells under varying pH conditions. At pH 6, we initially detected a significant release of H_2_, with 7.6 mL H_2_/L_medium_ generated on day 1. However, this H_2_ production gradually declined, reaching only 1.2 mL H_2_/L_medium_ by day 4. In contrast, at pH 8, we observed a higher initial H_2_ production, with 18.9 mL H_2_/L_medium_ generated on day 1, which decreased to 3.5 mL mL H_2_/L_medium_ by day 3. Notably, no hydrogen was detected after day 4 at pH 6 and after day 5 at pH 8. Throughout the experiment, pH 7 exhibited no trace of hydrogen production. These trends indicate a clear pH dependency in the metabolic processes driving hydrogen production in *O. alaskensis* G20.

At pH 6, the high availability of H^+^ ions favors the forward reaction of H_2_ evolution, which is catalyzed by periplasmic hydrogenases in *Desulfovibrio* species via the following chemical reaction (57, 58):


$$2H^{+}+2e^{-}↔H2$$

Under acidic conditions, the thermodynamic favorability of this reaction is enhanced due to the high proton gradient, as hydrogenases are most active in slightly acidic environments (53, 59). However, as hydrogen accumulates, the increasing redox potential creates feedback inhibition on hydrogenase activity (60, 61). Furthermore, the presence of sulfate as an electron acceptor in the medium allows for the reduction of sulfate to sulfide via the dissimilatory sulfate reduction pathway:

$SO_{4}^{2-}+4H_{2}+H^{+}\to HS^{-}+4H_{2}O$ (62, 63).

Under acidic pH, the undissociated H₂S begins to accumulate in the system (64). H₂S is known to inhibit hydrogenase enzymes by binding to the active sites, thereby limiting further H_2_ production (65). This mechanism explains the sharp decline in H_2_ production observed after day 1. At pH 8, the initial hydrogen production was significantly higher than at pH 6, suggesting improved enzyme stability under alkaline conditions. Hydrogenases in *Desulfovibrio* are known to retain activity in mildly alkaline environments, as evidenced in studies where hydrogen evolution increased under similar conditions (66). However, the reduced concentration of H^+^ at higher pH levels acts as a limiting factor, slowing the rate of H_2_ evolution. Additionally, the dissociation of H_2_S into HS^−^ and H^+^ under alkaline conditions reduces the inhibitory effects of H_2_S, which may initially allow higher H_2_ production (63). The decline observed by day 3 may be attributed to substrate limitation and the eventual exhaustion of electron donors required to sustain hydrogenase activity. The absence of hydrogen production at pH 7 can be explained by a thermodynamic equilibrium between H_2_ evolution and consumption. At neutral pH, the hydrogenase-catalyzed reaction reaches a state where the rate of H_2_ production is balanced by its consumption in cellular processes, such as sulfate reduction or proton reduction. This balance prevents any net accumulation of H_2_ in the medium. Therefore, the proposed reaction below may proceed more slowly at neutral pH due to reduced proton availability and thermodynamic constraints.

$CH_{3}CHOHCOOH+2\;H_{2}O\to CH_{3}COOH+H_{2}CO_{3}+2\;H_{2}$ (67).

As a result, neither the forward nor the reverse reactions dominate, further reinforcing the lack of detectable H_2_ production.

The interplay between pH and H^+^ availability highlights the complexity of H_2_ metabolism in *O. alaskensis* G20. At acidic pH, favorable thermodynamics drive H_2_ production; however, it is gradually inhibited by the accumulation of undissociated H_2_S, which disrupts hydrogenase activity. At alkaline pH, enhanced enzyme stability initially supports higher H_2_ production, but H^+^ limitation and substrate depletion eventually reduce the production rate. At neutral pH, the system reaches a state of metabolic and thermodynamic equilibrium where H_2_ evolution and consumption are balanced, resulting in no net hydrogen production. Overall, the trends observed across varying pH conditions demonstrate how pH-dependent processes regulate H_2_ metabolism in *O. alaskensis* G20. This hydrogen production can be correlated with the expression profile of NiFeSe and *hyd* hydrogenases.

In line with our findings, the expression profile of NiFeSe exhibited consistent upregulation in both pH 6 (log2FC = 2.5) and pH 8 (log2FC = 2.6) during the growing phase. However, as *O. alaskensis* G20 transitioned into the stationary phase, a downregulation of NiFeSe (log2FC = −0.6) was evident in pH 6. This suggests that the cellular environment had reached a pH equilibrium of around 7, with no further proton adjustments required. Conversely, in pH 8, we observed a slight, but insignificant, upregulation (log2FC = 0.2) of NiFeSe, coinciding with the absence of detectable hydrogen production by day 4 in the stationary phase. The expression levels of *hyd* were notably upregulated at pH 6 during the growing phase (log2FC = 1.8) and also at pH 8 (log2FC = 0.5). By the stationary phase, *hyd* remained upregulated at pH 6 (log2FC = 1.1), whereas at pH 8, the upregulation was minimal with a log2FC of 0.1, which was not statistically significant.

#### Central carbon metabolism

In our study, we observed that the expression of lactate dehydrogenase (LDH) was downregulated at pH 6 (log2FC = −2.9) during the exponential growth phase. However, as the culture entered the stationary phase, LDH expression was upregulated (log2FC = 2.6), coinciding with a noticeable increase in lactate consumption rate (days 3–5). Notably, we observed a pH shift during the stationary phase, where pH 6 approached pH 7 (day 4). This indicates that the metabolic activity of the cells still relies on lactate as an electron donor at pH 6, while lactate consumption is diminished at pH 7 after day 4. These findings align with earlier research by Arriaga et al., which explained the allosteric or homotropic effects of acidic pH on LDH (68). During the growing phase, we observed an upregulation (log2FC = 4.9) of LDH at pH 8. However, during the stationary phase, a lesser fold change was observed (log2FC = 2.0), which may be possibly due to the pH level approaching that of pH 7 by day 3. At pH 8, no significant lactate consumption was observed beyond day 4. This phenomenon is supported by the study conducted by Stolyar et al., which demonstrated that lactate consumption ceased abruptly following the pH shift (30).

The pyruvate formate-lyase 1-activating enzyme (PFL), crucial for converting pyruvate to acetyl-CoA and formate in anaerobic metabolism (69), showed downregulation at pH 6 (log2FC = −2.6), indicating reduced formate production compared to pH 7. Conversely, upregulation was observed at pH 8 (log2FC = 1.4), likely increasing formate production to counterbalance the alkaline conditions. During the stationary phase, PFL remained significantly downregulated at pH 6 (log2FC = −2.4) and upregulated at pH 8 (log2FC = 1.4). This suggests that at pH 8, where the cells adapted to alkaline conditions, formate production was induced, while no significant change in expression fold was observed between the two phases at pH 6 and pH 8.

FDH, responsible for converting formate to carbon dioxide (70), showed downregulation at pH 6 during the growing phase, likely due to reduced metabolic flux from low formate production under acidic conditions. At pH 8 during the growing phase, the modest upregulation of FDH (log2FC = 0.4) suggests minimal formate conversion within the cell, potentially allowing formate to be transported unchanged to counteract alkaline conditions. During the stationary phase, the continuous downregulation of formate dehydrogenase at pH 6 and upregulation at pH 8 indicates that formate production is less crucial for generating additional hydrogen ions (one ion per mole of products) at this growth stage. Therefore, FDH may not contribute significantly to establishing a proton gradient at pH 6, unlike at pH 8 where its expression level is comparable to that at pH 7.

Pyruvate carboxylase (PC) is responsible for combining pyruvate and carbon dioxide (generated from the action of FDH) to form oxaloacetate (71), a key step in the incomplete/reductive TCA cycle of *O. alaskensis* G20 and other SRB. This gene showed downregulation at the growing phase with log2FC = −1.8 at pH 6, while showing no significant fold change at pH 8 (log2FC = 0.07). This downregulation at pH 6 may be attributed to the extracellular release of cycle intermediates to counteract acidic conditions. At the stationary phase, PC remained downregulated (log2FC = −2.1) at pH 6, whereas at pH 8, it was significantly upregulated (log2FC = 2.3), potentially reflecting a response to alkaline conditions to support metabolic activity.

#### Cell division coordinator

The process of bacterial cell division involves a coordinated sequence of events, starting from chromosome replication and segregation and involved in septation. In order to understand how alterations in pH affect cell division, we conducted gene expression studies focusing on genes within the *fts* locus. Among these genes, *ftsZ*, *ftsQ*, and *ftsA* play major roles. *ftsZ*, a key component of the Z-ring protein structure localized at the division site, plays a vital role in coordinating cell division and promoting septum formation. The expression of the *ftsZ* gene is influenced by various factors, including environmental conditions and intracellular signaling. However, it is noteworthy that under many stress conditions, such as copper exposure, temperature changes, and acidic pH stress, the expression of the *ftsZ* gene tends to be downregulated (18, 72, 73).

In this study, the *ftsZ* gene exhibited downregulation by 2.4- and 1.8-fold in acidic and alkaline conditions, respectively, during the exponential growth phase. Moreover, in the stationary phase, *ftsZ* remained downregulated by 2.1-fold in acidic conditions but showed upregulation by 1.2-fold in alkaline pH. Once the *Z*-ring is established, *ftsZ* plays a crucial role in recruiting *ftsA* to the septum (74). The balance between *ftsA* and *ftsZ* levels determines the initiation of cell division, with *ftsA* inhibiting the process while *ftsZ* promotes it. Interestingly, transcription of the *ftsA* gene was induced under acidic conditions, while *ftsZ* expression was repressed in response to acidity (72). During the growing phase, the *ftsA* gene was consistently downregulated in both pH 6 (log2FC = −1.9) and pH 8 (log2FC = −0.9), with sustained downregulation observed at the stationary phase in both pH 6 (log2FC = −2.1) and pH 8 (log2FC = −0.2). *FtsQ* is a pivotal component of the bacterial divisome essential for septal ring formation, interacting with other divisome proteins, precise localization to the division site, coordination of peptidoglycan synthesis and remodeling, regulation of cell division timing, and overall bacterial viability (75). This gene exhibited downregulation (log2FC = −3.2) and upregulation (log2FC = 0.9) in pH 6 and pH 8, respectively, during the growing phase; notably, *ftsQ* maintained similar patterns in pH 6 (log2FC = −0.2) and pH 8 (log2FC = 1.3) during the stationary phase, indicating significant variation in *ftsQ* expression in acidic conditions between the stationary and growing stages. This highlights the sensitivity of cell division to different stressors, including alterations in pH.

#### Amino acid synthesis

At low external pH, specific enzymes involved in amino acid catabolism, such as amino acid decarboxylases, generate amines that are exported, effectively reversing acidification (76). Additionally, in the presence of alkaline stress, amino acid consumption can lead to cytoplasmic protonation, minimizing the impact of alkaline conditions (4, 77). Enzymes associated with amino acid synthesis, such as o-acetylserine sulfhydrylase A (*CysK*) and tryptophan deaminase (*tnaA*), are induced under alkaline conditions (78). In bacteria like *D. vulgaris*, gene expressions related to amino acid metabolism are moderately upregulated (30). Examples include the upregulation of genes encoding tryptophan synthase subunits, biosynthesis and transport of amino acids, and key enzymes involved in the production of cysteine, lysine, leucine, and other amino acids (4, 79). However, the genome search did not identify the *tnaA* gene within *O. alaskensis* G20; therefore, *CysK* and glutamine synthetase (GLS) were selected for expression studies, which have a critical role in acid-base balance.

In this study, during the growing phase, *CysK* exhibited significant downregulation (log2FC = −2.1) at pH 6 and upregulation (log2FC = 2.2) at pH 8, whereas at the stationary phase, *CysK* remained downregulated (log2FC = −1.0) at pH 6 and remarkably upregulated (log2FC = 6.0) at pH 8. This phenomenon can be attributed to the role of *CysK* in producing ammonia and acetate as products from 0-acetylserine, as reported in studies showing higher expression under alkaline conditions and downregulation under acidic conditions (80), which aligns with our findings during the growing stage where the acidic pH remained below 7 by day 2. Additionally, GLS showed downregulation in both pH 6 (log2FC = −3.8) and pH 8 (log2FC = −1.9) during the growing phase, followed by upregulation at pH 6 (log2FC = 0.2) and pH 8 (log2FC = 3.8) during the stationary phase. Studies in *Escherichia coli* have identified two groups of GLS with distinct pKa values of 7.1 and 8.2, indicating specific pH optima for their function (81). The downregulation of GLS at both acidic and alkaline pH levels suggests that the GLS responsible for converting ammonia and glutamate to glutamine operates optimally at neutral pH, which aligns with the stationary phase conditions where pH 6 and pH 8 converge toward pH 7.

#### *F*_0_–*F*_1_ ATPase

The ATP synthase complex comprises two components: *F*_0_ and *F*_1_. The *F*_0_ component spans the membrane, forming a proton channel, while the *F*_1_ component, extending into the cytoplasm, synthesizes ATP (82–84). During oxidative phosphorylation, the proton gradient across the membrane drives *F*_0_ rotation, inducing conformational changes in *F*_1_ that enable ATP synthesis from ADP and Pi (85, 86). The *F*_0_*–F*_1_ ATPase operates bidirectionally—synthesizing ATP or hydrolyzing it to pump protons—depending on the electrochemical gradient (87–89). Its activity is tightly regulated by factors such as ADP/Pi availability, the proton gradient, and cellular energy demands (90).

In our study on *F*_0_*–F*_1_ ATPase expression, we focused on the following *F*_1_ genes: *atpA* (encoding the alpha subunit), *atpH* (encoding the beta subunit), and *atpD* (encoding the delta subunit). Additionally, we analyzed *atpB*, which encodes the subunit a of *F*_0_. At pH 6, all genes were significantly downregulated during both the growing (*atpA*: −1.9, *atpH*: −4, *atpD*: −2.2, and *atpB*: −0.7) and stationary phases (*atpA*: −9.2, *atpH*: −3.9, *atpD*: −4.2, and *atpB*: −2), reflecting severe ATP energy stress under acidic conditions. At pH 8, during the growing phase, all genes*—atpB* (−0.2), *atpH* (−0.9), and *atpD* (−0.1)—showed downregulation, except for *atpA* (0.7), signifying insignificant gene expression for all ATPase genes. During the stationary phase, *atpB* (1.8) and *atpD* (0.7) were upregulated, indicating an adaptive response to maintain ATP synthesis, while *atpA* remained strongly downregulated (−7.1), and *atpH* was insignificantly downregulated (−0.2).

Notably, in *E. coli*, the *atpABCEFGI* operon is known to be downregulated under acidic conditions, whereas in *D. vulgaris*, a previous study reported upregulation of *atpA*, *atpB*, and *atpH* genes in response to low pH (39, 91). The proton pumps in bacterial cells generally function to expel protons outward, maintaining a negative membrane potential across a wide range of external pH conditions (92). This outward proton flow is crucial for preserving pH homeostasis, especially under acidic conditions where an excess of protons in the cytoplasm can inhibit ATP hydrolysis (93). The *F*_0_*–F*_1_-ATPase enzyme plays a critical role in this regulation by hydrolyzing ATP to drive the export of protons from the cytoplasm, thereby preventing internal acidification (94). When the proton motive force drops below a critical threshold—often triggered by decreasing extracellular pH—*F*_0_*–F*_1_-ATPase may switch to function as a proton pump (95). This switch is believed to result from the reorientation of the ε subunit (an endogenous ATPase inhibitor, *atpH*) toward the *F*_0_ component and away from the β subunit of *F*_1_ (4). This conformational rearrangement enables ATP hydrolysis, facilitating the active export of protons to maintain pH balance. In *O. alaskensis* G20, the significant downregulation of *F*_0_*–F*_1_-ATPase genes (e.g., *atpA*, *atpB*, *atpD*, and *atpH*) at pH 6 suggests that under acidic stress, the cells aim to conserve energy by minimizing ATP hydrolysis. This response likely reflects a strategy to avoid further proton influx, as the activity of ATP synthase could exacerbate cytoplasmic acidification. At pH 8, during the stationary phase, the upregulation of specific genes like *atpB* and *atpD* indicates a shift toward maintaining ATP synthesis and stabilizing the proton gradient. This adaptive response helps counteract the low proton availability under alkaline conditions, ensuring essential cellular functions can proceed.

#### Transporters

Na^+^/H^+^ antiporters are essential membrane proteins involved in maintaining H^+^ and Na^+^ homeostasis, pivotal for intracellular pH balance, cellular Na^+^ levels, and cell volume regulation. *NhaA*, that codes for Na^+^/H^+^ antiporter, exchanges Na^+^ for H^+^ across membranes. During the growing phase, *NhaA* was downregulated at pH 6 (log2FC = −2.4) and showed a non-significant downregulation at pH 8 (log2FC = −0.3). Similarly, in the stationary phase, *NhaA* was downregulated at pH 6 (log2FC = −1.7) and upregulated at pH 8 (log2FC = 1.6). Studies by Padan et al. demonstrated that *E. coli NhaA* is inactive below pH 6.5 but shows a significant increase in activity at alkaline pH, peaking at pH 8.5 (96). Sakuma et al. found that a mutant expressing only *NhaA* effectively extrudes sodium ions across a broad pH range from 6 to 9 (97). In contrast, Shijuku et al. reported lower expression of *E. coli NhaA* under alkaline conditions (98). Additionally, Pinner et al. observed no growth in the presence of 100 mM NaCl at pH 8.5 when *nhaA* was deleted, indicating reduced sodium ion extrusion activity at alkaline pH (99). These findings collectively underscore the critical role of Na+/H + antiporters, particularly *NhaA*, in alkaline conditions in *O. alaskensis* G20, aligning with previous studies highlighting their importance in pH adaptation and ion homeostasis.

The cytochrome c3 hydrogenase (encoded by *cyh* gene), known for its electron transfer activity and heme ligand binding ability in *D. vulgaris*, displays distinct behavior under extreme pH conditions. Protonation of the axial ligands can generate high-spin forms of cytochrome c3, influencing spin equilibrium (low spin/high spin). Santos et al. reported spin alterations at acidic pH and the consequential changes in redox potentials associated with these modifications (100). In our study, *cyh* showed downregulation at pH 6 during both the growing phase (log2FC = −0.8) and stationary phase (log2FC = −0.7), while exhibiting consistent upregulation at pH 8 in both stages (log2FC = 0.4). In a study by Matsuno et al., cytochrome c showed a 3.6-fold higher expression in alkaline conditions (pH 10) in *Pseudomonas alcaliphila* AL15-21 as compared to pH 7. This upregulation may reflect a response to optimize electron transfer activity and heme ligand binding under alkaline pH environments. The contrasting behavior of *cyh* at acidic vs alkaline pH conditions aligns with the notion that the protonation of ligands can affect the spin state and redox properties of cytochrome c3, as reported in previous studies.

HKs serve as pivotal regulators in bacterial two-component systems, orchestrating cellular responses to environmental cues. The majority of HKs are homodimers with autokinase, phosphotransfer, and phosphatase activities. Notably, Liu et al. reported a pH-mediated conformational switch in an HK from *Thermotoga maritima* that inhibits its phosphatase activity under acidic conditions (101). In our study, the observed downregulation of HK at pH 6 during both growing (log2FC = −3.1) and stationary stages (log2FC = −3.3) suggests a potential pH-dependent regulation of HK expression. Conversely, we noted a non-significant upregulation of HK at pH 8 in both growth stages (log2FC = 0.3). The downregulation at acidic pH aligns with findings of pH-mediated conformational changes affecting HK activity reported in other bacterial systems, suggesting a conserved regulatory mechanism across different organisms.

The genes *abcB* and *abcP* encode transporters for branched-chain amino acids and polar amino acids, respectively, located in the membrane subunit. During the growing stage, *abcB* and *abcP* were both downregulated at pH 6 with log2FCs of −0.9 and −4.2, respectively. At pH 8, *abcB* was upregulated (log2FC = 0.5), while *abcP* was downregulated (log2FC = −1.0). In the stationary phase, we observed downregulation of *abcB* (log2FC = −0.9) and *abcP* (log2FC = −1.7) at pH 6, whereas both genes were upregulated at pH 8 with log2FCs of 2.2 and 3.8, respectively. This suggests that under acidic pH conditions, there is minimal export of amino acids into the cellular environment, whereas export may occur at pH 8 to counterbalance the presence of hydroxide ions.

The PAS sensor system (Per-Arnt-Sim) plays a role in maintaining redox potential both inside and outside the cellular environment. Studies, such as those by Machmann et al., have demonstrated energy sensing based on the low potential of PAS couplings in *E. coli* related to Aer-PAS proteins, although specific regulatory mechanisms have not been extensively studied (102). Machmann et al. reported conformational shifts in the PAS domain triggered by the oxidized FAD (FAD_ox_)/FAD anionic semiquinone (FADASQ) redox couple. In our study, we observed downregulation of the PAS gene (log2FC = −2.0) at pH 6 during the growing phase, with no significant regulation observed in the stationary phase. At pH 8 in the growing stage, there was a substantial downregulation (log2FC = −4.2) of the PAS gene, whereas an opposite observation with significant upregulation (log2FC = 3.8) was noted at the stationary stage. The complex nature of the PAS domain makes it challenging to infer its specific role under extreme pH conditions, but the higher log2FCs observed suggest a strong significance and potentially unique involvement in pH homeostasis through redox potential regulation.

### Quantification of extracellular polymeric substances under variable pH

**Fig 5:**
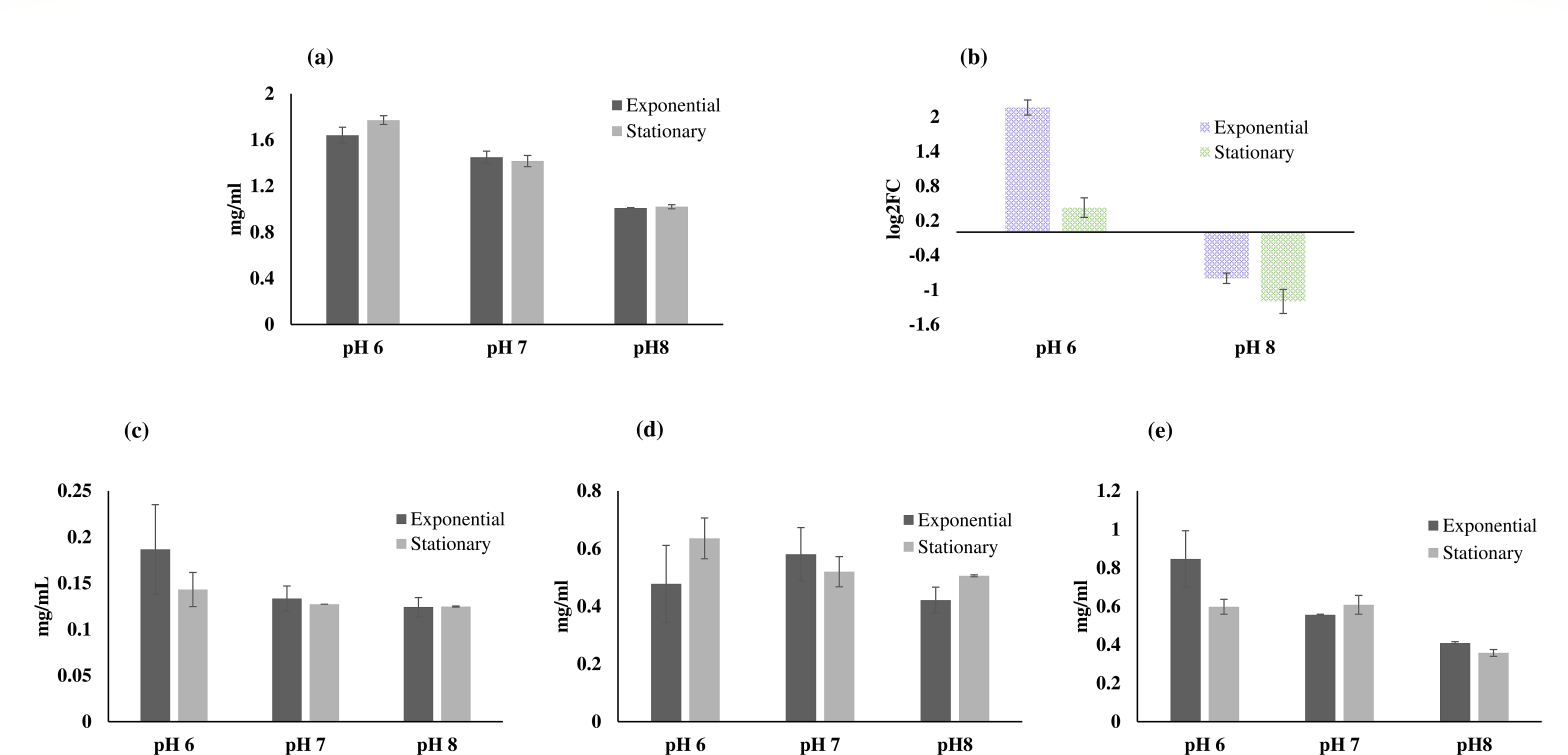
Illustration of the (a) total EPS yield (mg/mL), (b) gene expression (log2FC) of PS gene (capsule polysaccharide biosynthesis gene) at exponential and stationary phase, (c) carbohydrate content (mg/mL), (d) protein content (mg/mL), and (e) nucleic acid content (mg/mL). The EPS data (**a and **c–e) are represented for pH 6, pH 7, and pH 8, and gene expression data (b) are given for pH 6 and pH 8 with respect to pH 7. The statistical analysis included the determination of SD from triplicate measurements, with error bars representing the variability.

The total EPS yield from the planktonic culture was measured across pH 6, pH 7, and pH 8 conditions (Fig. 5a). The results showed that pH 6 yielded the maximum EPS during both growing and stationary phases (1.6 and 1.8 mg/mL, respectively), followed by pH 7 (1.4 and 1.5 mg/mL, respectively) and then pH 8 (1.0 mg/mL, in both stages). Notably, the EPS yield at pH 8 remained consistent between the exponential and stationary phases. In terms of gene expression related to PS (capsule polysaccharide biosynthesis gene), comparisons between pH conditions revealed distinct patterns (Fig. 5b). During the growing phase, the expression of the PS gene was significantly upregulated at pH 6 (log2FC = 2.0) compared to pH 7, while the upregulation at the stationary phase was lower (log2FC = 0.4). However, at pH 8, both growing and stationary phases exhibited downregulation of the PS gene, with log2FC values of −0.8 and −1.1, respectively.

These gene expression patterns align with the observed EPS yield results, where pH 6 consistently produced the highest EPS yield, and pH 8 yielded the least EPS. The upregulation of the PS gene at pH 6 during the growing and stationary phase likely contributes to increased EPS production. The downregulation observed at pH 8 in both phases suggests a shift in metabolic priorities or environmental adaptation, leading to reduced EPS production.

Analysis of EPS composition in terms of carbohydrates, proteins, and nucleic acids indicated that the EPS of *O. alaskensis* G20 primarily consists of a proteinaceous component in neutral pH (Fig. 5c through e). However, notable differences were observed in the fractions of carbohydrates, proteins, and nucleic acids between pH 6 and pH 8 when compared to neutral pH conditions. In terms of carbohydrate content, pH 6 exhibited higher levels during the exponential phase (0.7 mg/mL) compared to the stationary phase (0.14 mg/mL), with a fraction of 13% ± 1.7%. Conversely, the carbohydrate content remained relatively consistent between the two stages for pH 7 (0.12 mg/mL) and pH 8 (0.11 mg/mL), with fractions of 9% ± 1.1% and 10.9% ± 0.8%, respectively. These findings suggest that pH 6 favors carbohydrate production compared to neutral pH conditions.

Regarding protein content, the fractions at pH 6, pH 7, and pH 8 were 38% ± 1.2%, 45% ± 1.5%, and 49.5% ± 0.6%, respectively. This indicates an increasing trend in protein fraction with increasing pH, with pH 8 exhibiting the highest protein content. Similarly, the fractions of nucleic acids differed significantly among the pH conditions, with pH 6, pH 7, and pH 8 showing fractions of 51% ± 1.1%, 37% ± 1.2%, and 49.5% ± 0.8%, respectively. These results suggest a pH-dependent regulation of nucleic acid content, with pH 6 and pH 8 showing higher nucleic acid fractions compared to pH 7. Overall, these observations imply that changes in environmental pH can influence the metabolic processes and cellular composition of *O. alaskensis* G20. pH 6 appears to promote carbohydrate production, while higher pH levels (pH 7 and pH 8) favor increased protein and nucleic acid fractions.

### Phenotypic changes of *O. alaskensis* G20 cells

**Fig 6:**
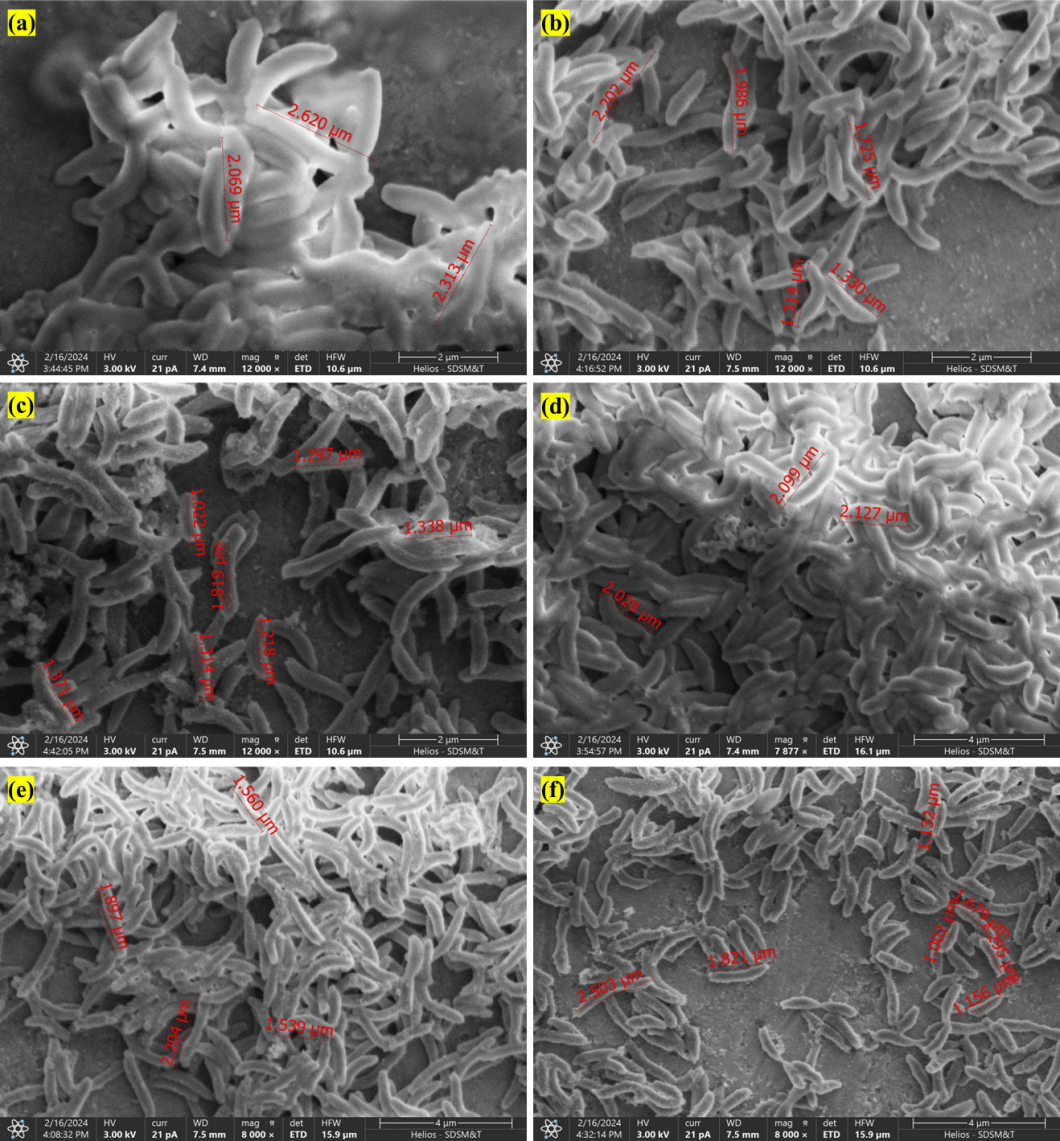
SEM images showing variation in cell size of *O. alaskensis* G20 at the growing stage under (a) pH 6, (b) pH 7, and (c) pH 8 and at the stationary stage under (d) pH 6, (e) pH 7, and (f) pH 8. Cell lengths are indicated by red measurement lines with corresponding numerical values. The SEM images pertained to growing and stationary stages and are scaled at 2 µm and 4 µm, respectively.

The SEM analysis was performed with pH 6, pH 7, and pH 8, as shown in Fig. 6. The observed variations in rectilinear cell length across different pH conditions provide insights into the pH-dependent effects on cell growth and morphology in *O. alaskensis* G20. During the growing phase, cells grown at pH 6 exhibited longer average lengths (ranging from 2 to 2.6 µm) compared to cells at pH 7 (ranging from 1.3 to 2.2 µm) and pH 8 (ranging from 1 to 1.4 µm). This suggests that acidic conditions (pH 6) may be more conducive to cell elongation and growth, potentially indicating optimal conditions for metabolic activity and nutrient uptake required for cell expansion. Conversely, cells at pH 8 displayed shorter lengths, indicating a possible limitation in cellular growth under alkaline conditions. As the culture progressed into the stationary phase, the trend in cell length differences persisted. Cells at pH 6 maintained the longest average length (~2 µm), followed by pH 7 (~1.6 µm) and pH 8 (~1.5 µm). This consistent pattern suggests that the influence of pH on cell size extends beyond active growth phases, reflecting sustained pH-dependent effects on cellular morphology and physiology throughout the growth cycle. Our previous studies on *O. alaskensis* G20 also showed cell elongation due to copper-induced stress (18, 27). Another report by Bosak et al. demonstrated the morphological variation of *O. alaskensis* G20 under phosphate-replete and phosphate-limited conditions, reporting cell lengths of 1.6 ± 0.3 µm at 200 µM phosphate and 2.2 ± 0.3 µm in cultures with an initial phosphate concentration of 2 µM (103). This signifies that *O. alaskensis* cells show variable cellular morphology under stress-related environments. The future study will implement machine learning algorithms to analyze full-frame SEM images of bacterial cells under stress, facilitating precise quantification and spatial mapping of morphological variations to uncover correlations with stress-induced responses.

The downregulation of *ftsZ* at pH 6 during both the growing phase and stationary phase suggests impaired initiation of cell division and potential elongation due to reduced expression of its counterpart, *ftsA*, which is essential for division termination. This observation is consistent with the SEM analysis under acidic conditions (pH 6), indicating a correlation between cell division and cell elongation. Conversely, in the stationary phase at pH 8, the observed upregulation of *ftsZ* and *ftsQ* (involved in divisome assembly and division site localization) suggests early initiation of cell division and enhanced site localization, which may contribute to shorter cell sizes under alkaline conditions. This response aligns with the SEM findings at pH 8, indicating a potential limitation in cellular growth but efficient division processes facilitated by increased divisome protein expression.

### Possible stress regulatory mechanisms of *O. alaskensis* G20 under acidic and alkaline conditions

**Fig 7:**
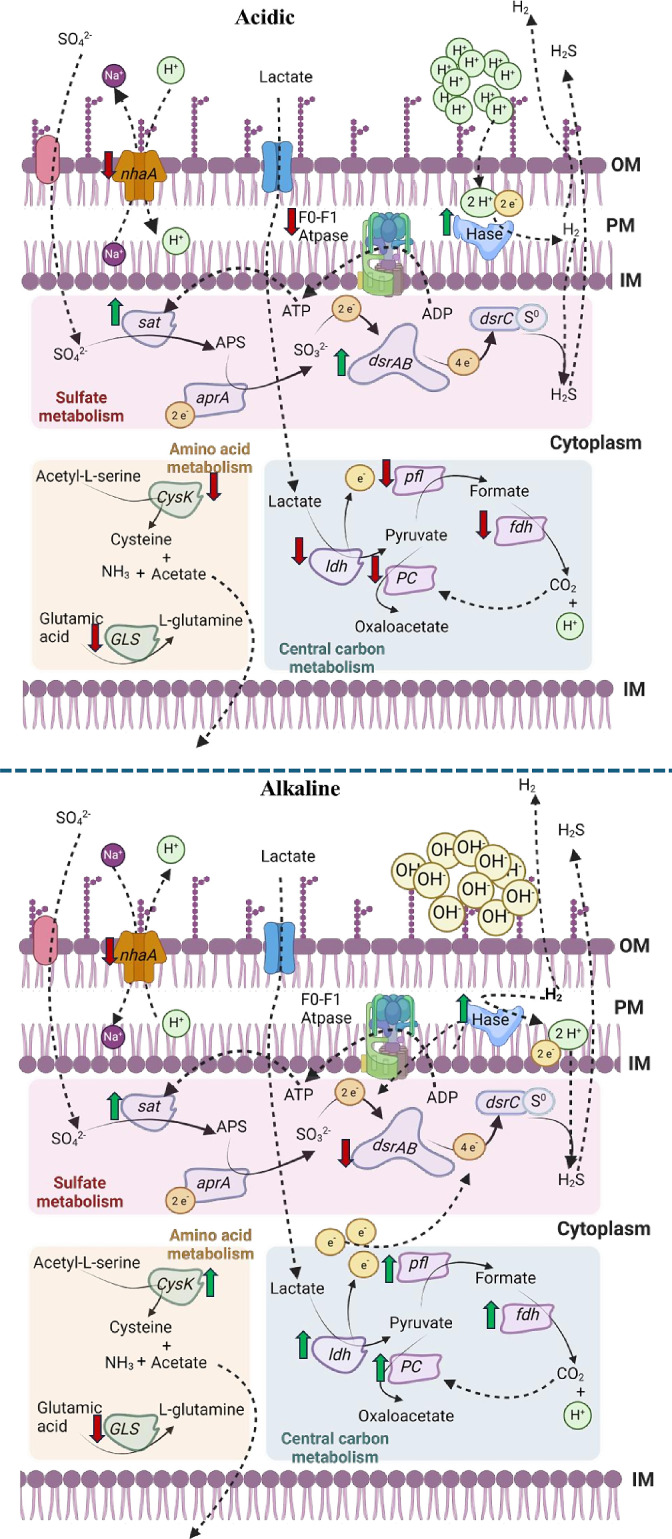
The overall putative pH stress regulatory mechanism in *O. alaskensis* G20 under acidic and alkaline conditions.

The concluded mechanism (Fig. 7) suggests that during the growing phase under acidic conditions, there was enhanced sulfate reduction activity and higher lactate consumption, whereas under alkaline conditions, there was downregulation of *dsrAB* genes, resulting in lower sulfate reduction and reduced lactate consumption. The concentration of H_2_S in its undissociated form is relatively high at pH 6, whereas at pH 8, the concentration of H_2_S in its undissociated form is relatively low. Kushkevych et al. showed the reduced dissimilatory sulfate reduction and lowered metabolic activity of *Desulfovibrio piger* Vib-7 when grown under acidic and alkaline stress conditions (25). The presence of a dissociated form of H_2_S would lead to elevated H^+^ ions, which help to balance out environmental OH^−^ ions, effectively neutralizing the pH. Furthermore, hydrogenases also play a crucial role in proton adjustments, where the initial amount of hydrogen production was significantly higher under alkaline conditions when compared to acidic conditions. This suggests that hydrogenase expression and hydrogen production in *O. alaskensis* G20 are closely linked to environmental pH and its impact on hydrogen equilibrium. Under center carbon metabolism, PFL and PC play an instrumental role to counterbalance the pH stress by reflecting adaptive formate production and supporting metabolic strategies to balance internal pH and support cellular metabolism. In our previous study on copper stress-associated transcriptomics analysis, *ftsZ*, *ftsQ*, and *ftsA* played a vital role in cell morphology by regulating cell division coordination and promoting septum formation (18). In this study, both the gene expression data and SEM analysis interlinked the role of *fts* operon in regulating cell morphology under alkaline and acidic stress conditions. *CysK* was significantly downregulated at pH 6 and upregulated at pH 8 during both the growing and stationary phases, indicating amino acid metabolism is crucial in maintaining pH homeostasis and its role in producing ammonia and acetate under alkaline conditions. Moreover, *F*_0_–*F*_1_ ATPase genes were downregulated at pH 6 during both growing and stationary phases, indicating a reduced ATP synthesis capacity under acidic conditions. At pH 8, these genes showed variable expression suggesting an adaptive response to optimize ATP synthesis and maintain proton gradients in different pH environments. Finally, the genes associated with transporters (*NhaA* and HK) indicate a reduced activity in ion exchange and signaling, potentially due to the inactivation of these proteins under acidic conditions. Conversely, at high pH, the upregulation of *NhaA* facilitates Na^+^/H^+^ exchange, aiding in maintaining ion homeostasis, while the upregulation of *Cyh* enhances electron transfer and heme ligand binding, optimizing cellular functions in alkaline environments. A related study by Tran and Stolyar observed similar gene expressions when *D. vulgaris* was grown under acidic artificial water and alkaline stress conditions, where significant expression of ATP synthase and antiporters was evident (26, 30). The upregulation of *abcB* and *abcP* transporters at high pH suggests a mechanism in which increased amino acid transport counterbalances excess hydroxide ions, facilitating cellular adaptation to alkaline stress.

### Conclusion

Our comprehensive analysis highlights the intricate genetic and metabolic adaptations of *O. alaskensis* G20 under varying pH conditions. Among the analyzed gene sets, significant fold changes were observed in genes located in the periplasmic and cytoplasmic regions, emphasizing their roles in proton exchange and electron generation to maintain pH homeostasis. Genes associated with sulfur metabolism were notably upregulated in acidic conditions to stabilize cytoplasmic pH but were significantly downregulated in alkaline environments due to the proton-consuming nature of the dissimilatory reduction pathway. To meet the increased energy demands at alkaline pH, genes involved in central carbon metabolism were significantly upregulated during the growing phase. This upregulation supported proton generation via *fdh* to sustain the dissimilatory reduction pathway and contributed to cytoplasmic pH neutralization. Conversely, under acidic conditions, these genes were downregulated due to the abundance of protons. In amino acid biosynthesis, the *cysK* gene exhibited significant upregulation under alkaline conditions, likely countering hydroxide ions through enhanced acetate production. Additionally, the Na^+^/H^+^ antiporter and PAS domain proteins were identified as critical for maintaining cellular functions under alkaline stress. Cell division regulation also exhibited pH-dependent modulation, as validated by the cell division coordinator *fts* operon. The expression of *ftsZ*, a key gene influencing cell division and cell length, was downregulated at pH 6 and upregulated at pH 8, resulting in different cell lengths. Furthermore, the EPS of *O. alaskensis* G20, primarily composed of proteinaceous fractions, showed the highest yield at pH 6, followed by pH 7 and pH 8. This suggests a strong link between pH and EPS production, potentially reflecting adaptive strategies to environmental stress. These findings collectively reveal the dynamic regulatory mechanisms employed by *O. alaskensis* G20 to adapt to fluctuating pH environments, balancing energy demands, proton gradients, and cellular integrity.

## Data Availability

The original contributions presented in the study are included in the article and its supplemental material.

## SUPPLEMENTAL MATERIAL

Table S1 aem.02565-24-s0001.docx Forward and reverse primers of gene sets used in expression studies.

ASM does not own the copyrights to Supplemental Material that may be linked to, or accessed through, an article. The authors have granted ASM a non-exclusive, world-wide license to publish the Supplemental Material files. Please contact the corresponding author directly for reuse.
