# Supplementary material for: pH-dependent genotypic and phenotypic variability in Oleidesulfovibrio alaskensis G20
Source: Appl Environ Microbiol. 2025 Mar 26;91(4):e02565-24. doi: 10.1128/aem.02565-24 (PMC12016547; doi:10.1128/aem.02565-24)
Supplement: Table S1 — Forward and reverse primers of gene sets used in expression studies. [file aem.02565-24-s0001.docx]

**pH-Dependent Genotypic and Phenotypic Variability in *Oleidesulfovibrio alaskensis* G20**

**Table S1**: Forward and reverse primers of gene sets used in expression studies.

| **Gene names** | **Gene Id** | **Protein Names** | | **Primer name** | **qPCR Primer sequence (5’🡪 3’)** |
| --- | --- | --- | --- | --- | --- |
| **Dissimilatory sulfate reduction** | | | | | |
| *dsrA* | Dde_0526 | Sulfite reductase, dissimilatory-type alpha subunit | | Forward  Reverse | ACCAGCCTGCAGGTAAGTTC  CGTGGGTCAGTTCCCAGAAA |
| *dsrB* | Dde_0527 | sulfite reductase, dissimilatory-type beta subunit | | Forward  Reverse | GGCTGAATCCGGCGAAAAAG  CGCAGAGCTTTCATGGCTTC |
| *sat* | Dde_2265 | sulfate adenylyltransferase | | Forward  Reverse | CGCTGTTGAAGTGTGTGACG  CGTAGTTCTGGCGGAAGGTT |
| **Hydrogenase** | | | | | |
| NiFeSe^1^ | Dde_2135 | Periplasmic (NiFeSe) hydrogenase, large subunit | | Forward  Reverse | ACCCCTTTAACCTTGTGGGC  TACAGACACCACCCTTTGCG |
| *hyd* | Dde_0081 | Periplasmic (Fe) hydrogenase large subunit | | Forward  Reverse | TGACGCAACCATAGACACCC  GTAGGCATAACGCAAAGCCG |
| **Central carbon metabolism** | | | | | |
| *ldh* | Dde_3604 | D-lactate dehydrogenase | | Forward  Reverse | AACAGGTCAGCCGCATAGTC  GCTCTCCGGTAATCACTCCG |
| *FdhA* | Dde_3513 | formate dehydrogenase, subunit alpha | | Forward  Reverse | CCACCTACAACGTGACGGAA  TGGCGATAGCCTCTACCCTT |
| *pfl* | Dde_3055 | pyruvate formate-lyase PFL | | Forward  Reverse | TGGATCAACTGCTGACCGAC  AGGAAGTTCCGGCTTTACGG |
| PC^1^ | Dde_1541 | Pyruvate carboxylase | | Forward  Reverse | GCGTGTCAACGACTTCAACC  AGCAATTTCCACGCGCTTTT |
| **Cell division coordinator** | | | | | |
| *FtsA* | Dde_1046 | Cell division protein FtsA | | Forward  Reverse | GCGACGACGAGGGAATAGAG  GATCGACCAGCGACAGGATT |
| *FtsQ* | Dde_1045 | Polypeptide-transport-associated domain protein FtsQ-type | | Forward  Reverse | CAGATAGACTCCGAAGCCGC  TGCCTGCTGTCAGGTGTATG |
| *FtsZ* | Dde_1047 | Cell division protein FtsZ | | Forward  Reverse | TTTATCACCGCCAACACCGA  CTTTCAAGGGCTGCCTGTCT |
| **Amino acid synthesis** | | | | | |
| *CysK* | Dde_3080 | Cysteine synthase | | Forward  Reverse | AGCATGATCGAATCGGCACT  TCATGGATTCCGGCATGGTC |
| GLS^1^ | Dde_0102 | Glutamine synthetase catalytic region | | Forward  Reverse | AAAAACCCTTTGCGGGCATC  GCAGCGCAGAAAACAAGGAA |
| **Fo-F1 Atpase** | | | | | |
| *atpA* | Dde_0987 | ATP synthase F1 sector subunit alpha | | Forward  Reverse | TGACAGACCGACGTTGATGG  GCCCTGCCCATCATTGAAAC |
| *atpB* | Dde_2700 | ATP synthase F0 sector subunit a | | Forward  Reverse | CTTTCGCTCTCTCTGCGTCT  ACCTTGGCCAGAGTGAACAG |
| *atpH* | Dde_0988 | ATP synthase F(1) sector subunit delta | | Forward  Reverse | TTCAATGCTGAAGAGCGGGT  TTTGCCATAGGGCAGAAGCA |
| *atpD* | Dde_0985 | ATP synthase F1 sector subunit beta | | Forward  Reverse | TGATGGAACCCTTGGTGGTG  GGACGTGCTGCTCTTTGTTG |
| **Transporters/ sensor protein** | | | | | |
| *NhaA* | Dde_2771 | Na(+)/H(+) antiporter NhaA | | Forward  Reverse | ATGACCAGAATGGCACCCAG  TGGTTCCCGCAGGCATTTAT |
| *Cyh* | Dde_3756 | Cytochrome-c3 hydrogenase | | Forward  Reverse | TTTCGGATTCACCCGCATCA  ACAAATCCAGCCACCGTCAT |
| HK^1^ | Dde_3717 | sensor signal transduction histidine kinase | | Forward  Reverse | TTTAAAACAGCCTGGCCCGA  CAGCGCAAATTCCAGCACAT |
| *abcB* | Dde_0159 | branched-chain amino acid transport | | Forward  Reverse | GTGCCTACGGCGGTACTTT  CATTGCCACGGCAAATGATG |
| *abcP* | Dde_0167 | Polar amino acid ABC transporter, inner membrane subunit | | Forward  Reverse | ATGACAACCAGCAGCGAAGT  CAGTCCATCAACACCGGACA |
| PAS^1^ | Dde_3740 | PAS sensor protein | | Forward  Reverse | TTCTGCACGATCTTTCCGCT  TCCTGCCGTTCTTCTTCCAC |
| **Flagellar biosynthesis** | | | | | |
| *flhA* | Dde_0380 | Flagellar biosynthesis protein FlhA | | Forward  Reverse | GTTCGCTCTGGATATGGGGG  TCGATACCCTTGATGCGGTG |
| *fliF* | Dde_0353 | Flagellar M-ring protein | | Forward  Reverse | ATGTCCATTCGCCCGTTTCT  CGGAAGACTCCAACCGTGAA |
| **Two-component** | | | | | |
| *luxR* | Dde_0977 | Two component transcriptional regulator, LuxR family | | Forward  Reverse | GATACCTGAACGACGCCAGA  ATCAGGTTGGAACGGTGCTT |
|  | Dde_2674 |  |  | Forward  Reverse | CGTTTTCCCCGCACAAAAGT  GTCGATGAGCGACTGGGAAA |
| *luxP* | Dde_3311 | Autoinducer 2-binding periplasmic protein LuxP | | Forward  Reverse | AAAGCATTCGTTCACCCCCA  GGCGCAGGCATATATCAGGT |
| **Polysaccharide synthesis** | | | | | |
| PS^1^ | Dde_3253 | Capsule polysaccharide biosynthesis protein | | Forward  Reverse | TCGCCGTGTTCGTGGTATTT  AAGCAGAAAGTCCATGCCGA |
| **Housekeeping gene** | | | | | |
| *recA* | Dde_2373 | Recombinase A | | Forward  Reverse | GATCGCCGAATGCCAGAAAC  TCGGCGATATCAAGTGCCTG |
|  |  |  |  |  |  |

^1^These are abbreviations to denote the genes for enhanced readability. Gene names are not annotated in databases.
